# INNOVATION IN AGING

**DOI:** 10.1093/geroni/igae098.0905

**Published:** 2024-12-31

**Authors:** Steven Albert

**Affiliations:** University of Pittsburgh, Pittsburgh, Pennsylvania, United States

## Abstract

N/A

